# Relationship between Maternal Central Obesity and the Risk of Gestational Diabetes Mellitus: A Systematic Review and Meta-Analysis of Cohort Studies

**DOI:** 10.1155/2020/6303820

**Published:** 2020-04-02

**Authors:** Da Yao, Qing Chang, Qi-Jun Wu, Shan-Yan Gao, Huan Zhao, Ya-Shu Liu, Yu-Ting Jiang, Yu-Hong Zhao

**Affiliations:** ^1^Department of Clinical Epidemiology, Shengjing Hospital of China Medical University, Shenyang, China; ^2^Clinical Research Center, Shengjing Hospital of China Medical University, Shenyang, China

## Abstract

**Objective:**

Nowadays, body mass index (BMI) is used to evaluate the risk stratification of obesity-related pregnancy complications in clinics. However, BMI cannot reflect fat distribution or the proportion of adipose to nonadipose tissue. The objective of this study is to evaluate the association of maternal first or second trimester central obesity with the risk of GDM. *Research Design and Methods*. We searched in PubMed, Embase, and Web of Science for English-language medical literature published up to 12 May 2019. Cohort studies were only included in the search. Abdominal subcutaneous fat thickness, waist circumference, waist-hip ratio or body fat distribution were elected as measures of maternal central obesity, and all diagnostic criteria for GDM were accepted. The random effect meta-analysis was performed to evaluate the relationship between central obesity and the risk of GDM.

**Results:**

A total of 11 cohort studies with an overall sample size of 27,675 women and 2,226 patients with GDM were included in the analysis. The summary estimate of GDM risk in the central obesity pregnant women was 2.76 (95% confidence interval [CI]: 2.35–3.26) using the adjusted odds ratio (OR). The degree of heterogeneity among the studies was low (*I*^2^ = 14.4, *P* = 0.307). The subgroup analyses showed that heterogeneity was affected by selected study characteristics (methods of exposure and trimesters). After adjusting for potential confounds, the OR of adjusted BMI was significant (OR = 3.07, 95% CI: 2.35–4.00).

**Conclusions:**

Our findings indicate that the risk of GDM was positively associated with maternal central obesity.

## 1. Introduction

Gestational diabetes mellitus (GDM) can be defined as different levels of abnormal glucose intolerance that occur for the first time during pregnancy [1]. It has affected 0.5–15% of pregnancies in the world and is one of the most common diseases of pregnancy [2]. Additionally, GDM is an important factor causing adverse pregnancy outcomes, which is hazardous for the mother and the newborn [3]. There are increased risks of eclampsia, preeclampsia, gestational hypertension, and future type 2 diabetes for the mother [4]. Several recent studies found several predisposing factors for GDM, such as age, obesity, and familial history of diabetes [5]. However, prepregnancy and maternal obesity are important factors, which also increases related pregnancy complications such as preeclampsia and fetal growth disorders [6].

There is a growing prevalence of maternal obesity worldwide [7]. Nowadays, body mass index (BMI) is used to evaluate the risk stratification of obesity-related pregnancy complications in clinics [8]. However, BMI cannot reflect fat distribution or the proportion of adipose to nonadipose tissue [9, 10]. Adipose tissue not only is a storage area for energy but also acts as an endocrine and immune organ that releases signals [11]. Therefore, excessive accumulation of adipose tissue affects body physiology, giving rise to chronic inflammatory responses and disarranging metabolic homeostasis. Hence, maternal central obesity is significant and can reflect fat distribution or the proportion of adipose to nonadipose tissue [12]. Existing research shows that maternal central obesity has many evaluation measures, such as waist circumference (WC)/waist-hip ratio (WHR) [13–16], abdominal subcutaneous fat thickness (SFT) [17–21], and body fat index (BFI) [22, 23], but the predictive value of these measures is not clear. The measurement accuracy and precision of WC are difficult to guarantee as it can only evaluate the condition of abdominal fat tissue, which has certain limitations [24, 25]. Maternal abdominal SFT and fat mass percentage (FMP) can be used as surrogate measures for maternal central obesity and are readily and accurately measured—they are quick, safe, and routinely used in pregnancy [23, 26].

Although some studies and meta-analyses have noted a relationship between BMI and GDM [27–30], there are no general studies and agreements about central obesity. An Australian longitudinal cohort study found that SFT was an important factor in determining obesity-related risk in pregnancy [31]. However, in the same year, a prospective cohort study found that subcutaneous adipose tissue depth was significantly associated with a higher risk for GDM in univariate analysis, but not after adjusting for covariates [20]. To collect the available information offering the best and most reliable sources of scientific evidence, we followed PICOS (participants, interventions, comparisons, outcome, and study design) guidelines. Hence, we systematically and comprehensively included the cohort studies and investigated the impact of maternal central obesity on the risk of GDM using a meta-analysis.

## 2. Research Design and Methods

The meta-analysis followed the recommendations of the PRISMA group. The meta-analysis was registered at PROSPERO on 31 July 2019, with registration number CRD42019137445.

### 2.1. Search Strategy

A search was conducted using PubMed, Embase, and Web of Science to find English-language medical literature published up to 12 May 2019. Our search comprised different keywords and Medical Subject Headings (MeSH) terms, and the search strategy for all literature databases includes “Obesity, abdominal” or “Obesity, abdominal” or “Waist circumference” or “Waist circuit” or “Waist-hip ratio” or “Body fat distribution” or “Body fat index” and “Pregnancy” or “Pregnant women” and “Diabetes, gestation” or “Gestational diabetes” or “Gestational diabetes mellitus” (Supplement [Supplementary-material supplementary-material-1]). At the same time, we contacted study authors when we needed to obtain additional information that was not available in the online publications or supplementary materials. In addition, we checked duplicate papers with NoteExpress software.

### 2.2. Inclusion/Exclusion Criteria

Cohort studies were included in the search by the following information: body fat distribution or central obesity as exposure variables and GDM as an outcome variable; women with information in the first or second trimester measurements (body fat distribution, WC, WHR, or SFT); women having been investigated for GDM during their pregnancy were eligible for inclusion.

We excluded non-English-language medical literature; women with previously diagnosed diabetes (type 1 or 2); studies that had not reported the odds ratio (OR), relative risk (RR), confidence interval (CI), and exposure measurement or inadequate data to calculate such values; and case reports, letter to editor and previous systematic reviews, and meta-analyses.

### 2.3. Data Extraction and Quality Assessment

In this study, all relevant publications were inserted in NoteExpress software and reviewed independently by two authors (YD and ZH). When two reviewers disagreed, the literature was resolved by a third reviewer (WQJ). Then, qualified papers were obtained for full-text screening. After the final evaluation, we extracted information including author's name, publication year, country, study design, study population, ethnicity, exposure measurement, GDM diagnosis criteria, matching/adjustment variates, and risk estimates and 95% CIs. All extracted data were then entered into Excel software. A total of 11 cohort studies with an overall sample size of 27,675 women and 2,226 patients with GDM were included in the analysis. To assess study quality, we used the Newcastle–Ottawa quality assessment scale (NOS) for cohort studies. In the meta-analysis, the NOS guideline-modified studies which achieved five or more stars were considered of high quality [32].

### 2.4. Statistical Analysis

The summary effect analysis was performed using Stata 11.2. After extracting and sorting the article data, we evaluated the ORs and 95% CIs for the highest level of maternal central obesity with those of the lowest level of maternal central obesity. Fixed and random effect models were applied to produce the summary estimates to determine the relationship between the exposure to maternal central obesity and the risk of GDM [33]. Higgins and Thompson's *I*^2^ was applied to determine the degree of heterogeneity between the studies [34]. The results were defined as highly heterogeneous for *I*^2^ > 50% [35]. We evaluated the possibility of publication bias using Begg's test, Egger's test, and a funnel plot of study effect size against standard error. We also used subgroup analysis including geographical location, number of participants, number of cases, method of exposure, trimester, GDM diagnostic criteria, and whether adjusted potential confounders in the analyses (e.g., age, ethnicity, BMI, family history of diabetes, parity, smoking, and education level). Statistical significance was defined as *P* < 0.05.

## 3. Results

### 3.1. Included Studies

**Figure 1:**
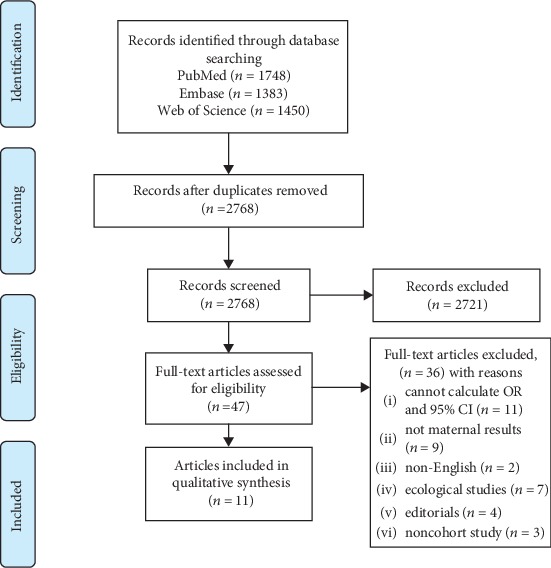
Flow chart of study selection.

Searching the three databases produced 4,511 potential studies. There were 1,743 duplicated studies and 2,721 excluded based on titles and abstracts. When checking the records and removing the duplicates, 47 were fully reviewed and 14 articles were identified that met inclusion criteria (Figure 1). In 47 articles reviewed in the full text, we excluded 11 articles because the articles cannot calculate OR and 95% CI and 9 articles were excluded because the outcome was not maternal results. The remaining 27 articles were excluded due to non-English (*n* = 2) or owing to ecological studies (*n* = 7), or editorials (*n* = 4), and noncohort study (*n* = 3). Finally, a total of 11 cohort studies with an overall sample size of 27,675 women and 2,226 patients with GDM were included in the analysis.

### 3.2. Characteristics and Quality Assessment of Included Studies

The baseline information of each included study is presented in Table 1. From the included studies, 2,226 and 25,449 pregnant women were GDM and non-GDM, respectively. A total of seven studies were from non-Asian countries [14, 16–18, 20–22], and four were from Asia [13, 15, 19, 23]. The GDM was diagnosed based on two methods among all the studies: five studies used Carpenter/Coustan diagnostic criteria [17–19, 21, 22], five studies used WHO screening criteria (75 g oral glucose tolerance test) [17–19, 21, 22], and one study used self-reports [16]. In these studies, nine studies reported the relationship between maternal central obesity and GDM in the first trimester [13–16, 18–21] and two reported the relationship in the second trimester [22, 23]. Six studies adopted WC or WHR [13–18], two used visceral adipose tissue depth (VAT) [20, 21], two used BFI or FMP [22, 23], and one used maternal SFT to measured maternal central obesity [19]. Most studies matched or adjusted for maternal age (*n* = 10) and BMI (*n* = 9). However, fewer studies adjusted for ethnicity (*n* = 6), family history of diabetes (*n* = 6), parity (*n* = 6), smoking (*n* = 4), and education level (*n* = 5).

The modified NOS method for evaluating article quality showed that 11 studies had five or more stars (Table 2).

### 3.3. Risk of GDM according to Maternal Central Obesity

**Figure 2:**
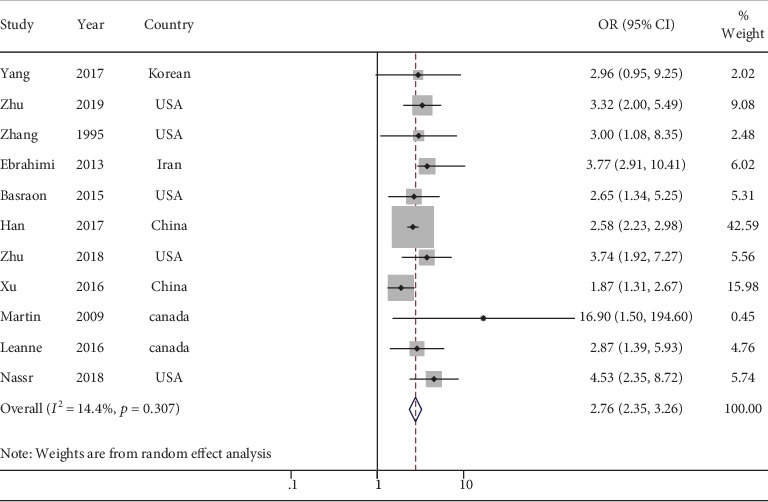
Forest plots (random effect model) of meta-analysis on the association between the concentration of central obesity and risk of GDM.

**Figure 3:**
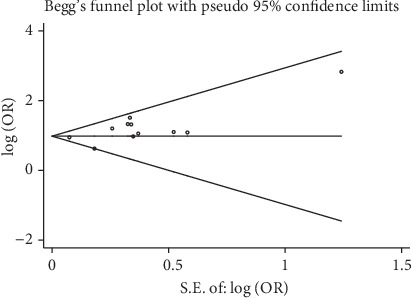
Funnel plot of included studies for potential publication bias.

We summarized the association between risk of GDM in pregnant women with maternal central obesity for the top and bottom levels of maternal central obesity (adjusted OR) (Table 3). A total of 11 studies provided adjusted OR for GDM: OR = 2.76 (95% CI: 2.35–3.26). The heterogeneity among studies was not significant (*I*^2^ = 14.4, *P* = 0.307) (Figure 2). Besides, Begg's test, Egger's test, and the funnel plot with 95% CI limits indicated no publication bias (*P* = 0.069) (Figure 3).

### 3.4. Subgroup Analysis and Sensitivity Analysis

The results of all subgroup analyses according to study characteristics are shown in Table 3. When we stratified by geographic location or number of participants, the OR for Asian countries and participants < 1000 was higher than that for others. When stratified using SFT, WC/WHR, BFI/FMP, and VAT to measure central obesity in pregnant women, the heterogeneity of BFI/FMP was significant (OR = 2.79, 95% CI: 1.18–6.59, *I*^2^ = 81.5, *P* = 0.020). After stratifying by trimester, the OR of the first trimester (OR = 2.73, 95% CI: 2.41–3.10) was similar to that for the second trimester group (OR = 2.79, 95% CI: 1.18–6.59). When stratified by assessment of outcome, the summarized OR of WHO screening criteria (OR = 3.76, 95% CI: 2.75–5.21) was higher than the Carpenter/Coustan diagnostic criteria (OR = 2.51, 95% CI: 2.14–2.95).

When adjusted for confounders, most ORs for factors showed slight decreases. However, for the factor of BMI in the risk of GDM, most studies (*n* = 9) adjusted for confounders, and the OR of adjusted BMI was significant (OR = 3.07, 95% CI: 2.35–4.00).

**Figure 4:**
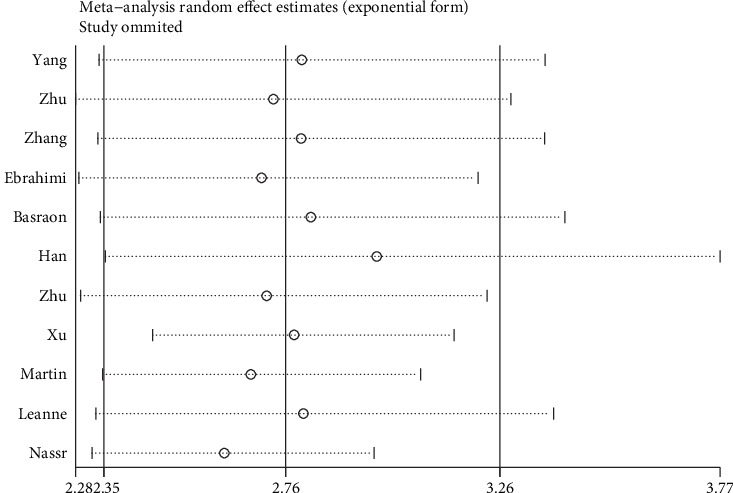
Sensitivity analysis of included studies.

We conducted a sensitivity analysis by omitting one included study at a time to investigate the influence of each study on the overall merged effect (Figure 4), and this showed that the meta-analysis results remained stable and reliable.

## 4. Discussion

Our meta-analysis of 27,675 pregnant women showed that maternal central obesity was directly associated with the risk of developing GDM. The adjusted OR of developing GDM was 2.76 for women with the highest level of central obesity compared with the lowest level.

This meta-analysis of 11 articles is the first study to offer convincing evidence of the relationship between increased maternal central obesity and the risk of GDM. Some meta-analyses have shown that the risk of GDM is directly related to prepregnancy BMI and pregnancy weight gain [27–30]. One study reported both the crude and adjusted ORs and RRs [30], and three other studies reported the crude and adjusted ORs or RRs of the effect of prepregnancy BMI (as a categorical variable) on the risk of GDM [36–38], which were adjusted for potential confounders, such as maternal age, ethnicity, family history of diabetes, education level, parity, and smoking status. After the adjustment of these GDM risk factors, these previous findings were consistent with our results. However, because BMI depends on the measurement of an individual's weight and height, it does not distinguish between bone, muscle, or fat mass and does not describe fat distribution [39, 40]. Hence, evaluating the relationship between increased maternal central obesity and the risk of GDM would be beneficial for assessing the effect of fat distribution [41].

In subgroup analyses, different measures differed only slightly, and VAT measured by ultrasound had the highest OR. This suggests that VAT may play an important role in GDM. As illustrated in existing studies, WHR and WC may be better predictors than BMI for cardiometabolic outcomes, including diabetes [42]. A cross-sectional study in Brazil indicated that WC of 86–88 cm was a good predictor of GDM for 20–24 weeks gestational women [43]. In this light, WC is a more practical measure for maternal central obesity, consistent with six of the studies included in our analysis. In addition, two included studies [22, 23] used BFI and FMP measured using bioelectrical impedance to evaluate maternal central obesity and had high heterogeneity (*I*^2^ = 81.5, *P* = 0.02). Of note, one study found a positive correlation between increasing FMP and the risk of GDM in overweight and obese women, and the OR increased 8.8-fold in the highest quartile of FMP compared with the lowest quartile [23]. Hence, further study is required to explore the different measurements of BFI. In terms of SFT and VAT, both are recognized as readily and accurately measured by ultrasound [23]. In a prospective case-control study in Italy involving women at 24–28 weeks gestation, increased VAT was a good predictor of GDM [44], as also detected in our study. This may indicate that VAT will emerge as an independent predictor of insulin resistance (IR), metabolic syndrome, and type 2 diabetes in nonpregnant populations [45]. Proposed mechanisms underlying the pathological association between VAT and IR include free fatty acid release into the hepatic portal circulation [46, 47]. Our results indicate that maternal central obesity, especially VAT, plays an important role in GDM.

Our subgroup analyses showed that a higher level of maternal central obesity had a similar GDM risk in the first and second trimester of pregnancy. A retrospective study provided some evidence that maternal central obesity at midpregnancy (18–22 weeks) was superior to BMI in identifying the risk of obesity-related pregnancy complications [48]. However, an Australian longitudinal cohort study found that SFT in 11–14 and 18–22 weeks were important factors determining obesity-related risk [31]. The subgroup results showed no significant differences among the trimesters. Hence, determining the central obesity of pregnant women during the first trimester may improve the efficiency of early screening of GDM in pregnant women.

According to previous studies, adipose tissue is a storage area for energy and acts as an endocrine and immune organ that releases signals [9, 10]. Excessive accumulation and different distribution of adipose tissue would give rise to chronic inflammatory responses and disarrange metabolic homeostasis [11]. Meanwhile, IL-37mRNA in the body is secreted by adipose tissue, and insulin sensitivity is related to whether IL-37mRNA can be secreted normally; the decrease in insulin sensitivity causes insulin dependence and resistance, leading to GDM [49]. Some experiments have studied the occurrence and development of IR in animal models. In mice treated with TNF-*α*, IR was positively correlated with the decrease of adiponectin and lipase [50]. However, determining the biological mechanisms responsible for the relationship between maternal central obesity and the risk of GDM will require further studies [51].

One of the strengths of this meta-analysis is that it is the first to evaluate the association between increased maternal central obesity and the risk of GDM and included a large number of GDM cases. All included studies were of high quality (all NOS scores > 5 stars). Another strength of this meta-analysis is that it included cohort studies, which decreased recall bias and provided a strong ability to test the etiological hypothesis and association.

This meta-analysis also had several limitations. First, one of the main limitations is a lack of levels of central obesity data. Although we identified many studies to assess the relationship between maternal central obesity and GDM, we also need a linear test to define linear or nonlinear relationships. Second, the included studies used varying measures for central obesity in women and GDM; thus, the summary estimates would not exactly reflect the same comparison for all studies. However, after performing subgroup analyses, the ORs for comparisons between different measures were fairly consistent among studies, suggesting that the definitions had no major effects on these findings. Third, various other risk factors may have contributed to the risk of developing GDM, such as ethnicity, BMI, family history of diabetes, parity, smoking, and several other variables. However, the included studies did not use multivariate analysis to adjust for all these potential confounders, which may result in confounding bias. In addition, when adjusting BMI for confounders, we found that the summarized OR of adjusted BMI was higher than nonadjusted BMI. Besides, the group of nonadjusted BMI was two article; it may influence the summarized OR. When adjusting for other confounders, the summarized OR of adjusted factors was lower than nonadjusted factors. The result indicated that the risk of GDM was positively associated with central obesity independently of BMI. Finally, this meta-analysis may have had inclusion criteria bias due to excluding non-English literature. In addition, because one included study used self-reporting of GDM, this may have introduced a response bias into our analysis.

## 5. Conclusions

Our findings indicate that the risk of GDM was positively associated with maternal central obesity. Therefore, this study can improve the efficiency of early screening of GDM for pregnant women in evaluating central obesity based on the measurement results. These findings can strengthen the scientific background for public health interventions for the control of the first or second trimester maternal central obesity independent of BMI.

## Figures and Tables

**Table 1:** Characteristics of studies included in the meta-analysis.

Author	Study design	Ethnicity	Study size	Period	GDM diagnosis	Matching/adjustment variates	Adjusted risk estimates (95% CI)	
De Souza et al., Canada, 2016 [20]	Prospective cohort study	Caucasian and non-Caucasian	52/485	First trimester	WHO	Maternal age, ethnicity, BMI, and family history of diabetes	VAT Q1	1.0
							VAT Q4	3.1 (1.1–9.5)
							TAT Q1	1.0
							TAT Q4	2.7 (1.1–7.8)
Martin et al., Canada, 2009 [21]	Prospective cohort study	White and non-White	16/62	First trimester	An abnormal 50 g glucose challenge test	Age and prepregnancy BMI	VAT Q1 < 4.74 cm	1
							VAT Q2 ≥ 4.74 cm	16.9 (1.5–194.6)
Yang et al., Korea, 2017 [19]	Prospective cohort study	Korean	41/333	First trimester	Carpenter and Coustan's diagnostic criteria	Prepregnancy BMI	SFT < 2.4 cm	1
							SFT ≥ 2.4 cm	2.96 (0.95–9.25)
Zhu et al., California, 2019 [17]	Prospective cohort study	Non-Hispanic White, Hispanic, African American, and other	186/1,750	First trimester	Carpenter and Coustan's diagnostic criteria	Age, ethnicity, prepregnancy overweight/obesity, family history of diabetes, previous GDM, preexisting hypertension, education, parity, and smoking	WC Q1	1
							WC Q4	2.84 (1.37–5.91)
							WHR Q1	1
							WHR Q4	3.82 (1.90–7.68)
Zhang et al., USA, 1995 [16]	Prospective cohort study	White and Black	44/720	First trimester	Self-reported	BMI, age, race, family history of diabetes in first degree relatives, parity, and fasting serum insulin	WHR 0.629–0.705	1
							WHR 0.706–0.742	2.28 (0.83–6.25)
							WHR 0.743–1.020	3.00 (1.08–8.35)
Ebrahimi-Mameghani et al., Iran, 2013 [15]	Prospective cohort study	Iran	41/948	First trimester	WHO	Age, education, BMI, and occupation	WC < 80 cm	1
							WC > 88 cm	3.77 (2.91–10.41)
Basraon et al., USA, 2015 [14]	Prospective cohort study	Hispanics, African Americans, White, and other	80/2,300	First trimester	WHO	Maternal age, education, race, weeks of gestation at enrollment, and alcohol and smoking status	WHR (normal)	1
							WHR (obese)	2.65 (1.34–5.25)
Han et al., China, 2017 [13]	Prospective cohort study	Chinese	1,383/17,803	First trimester	WHO	Maternal age, height, family history of diabetes, gestational weeks, parity, education, race, nonsingleton pregnancy, systolic blood pressure at registration, weight gain per week from registration to glucose challenge test, and smoking and drinking status	WC < 78.5 cm	1
							WC ≥ 78.5 and <85 cm	1.53 (1.31–1.78)
							WC ≥ 85 cm	2.58 (2.23–2.98)
							WC (BMI) < 78.5 cm	1
							WC (BMI) ≥ 78.5 and <85 cm	1.18 (1.00–1.40)
							WC (BMI) ≥ 85 cm	1.60 (1.345–1.91)
Zhu et al., California, 2018 [18]	Prospective cohort study	Non-Hispanic White, Hispanic, African American, Asian/Pacific Islander, and other	186/1,750	First trimester	Carpenter and Coustan's diagnostic criteria	Age, ethnicity, prepregnancy overweight/obesity, family history of diabetes, previous GDM, preexisting hypertension, education, parity, and smoking	WHR < 0.85	1
							WHR ≥ 0.85	3.74 (1.92–7.27)
Nassr et al., European, 2018 [22]	Prospective cohort study	USA	43/389	Second trimester	Carpenter and Coustan's diagnostic criteria	Age, BMI, history of diabetes, family history of diabetes current gestational hypertension or preeclampsia, subcutaneous fat ≥ 13 mm, PF ≥ 12 mm, and BFI < 0.5	Subcutaneous fat ≥ 13 mm	4.63 (1.60–13.38)
							Preperitoneal ≥ 12 mm	3.32 (1.06–10.42)
							BFI > 0.5	6.24 (1.86–20.96)
Xu et al., China, 2016 [23]	Prospective cohort study	Chinese	154/1,135	Second trimester	WHO	Pregnant age, alcohol, gravidity, prepregnant body weight, and prepregnant BMI	FMP < 28.77	1
							FMP > 35.01	1.95 (1.15–3.30)

CI, confidence interval; NA, not available; RR, relative risk; BMI: body mass index; WHO, World Health Organization diagnostic criteria; SFT, subcutaneous fat thickness; WC/WHR, waist circumference/waist-to-hip ratio; BFI/FMP, body fat index/fat mass percentage; VAT, visceral adipose tissue depth.

**Table 2:** Quality assessment of the cohort and cross-sectional studies included in the meta-analysis using the Newcastle–Ottawa Scale (NOS).

Study ID	Selection				Comparability	Outcome		
	Representativeness of the exposed cohort	Selection of the nonexposed cohort	Ascertainment of exposure	Demonstration that outcome of interest was not present at start of study	Comparability of cohorts on the basis of the design or analysis	Assessment of outcome	Was follow-up long enough for outcomes to occur	Adequacy of follow-up of cohorts
Yang et al., Korea, 2017 [19]	☆	☆	☆	☆	☆☆	☆	☆	☆
Martin et al., Canada, 2009 [21]	☆	☆	☆	☆	☆	☆	☆	☆
Yang et al., Korea, 2017 [19]	☆	☆	☆	☆	☆☆	☆	☆	☆
Zhu et al., California, 2019 [17]	☆	☆	☆	☆	☆☆	☆	☆	☆
Zhang et al., USA, 1995 [16]	☆	☆	☆	☆	☆		☆	☆
Ebrahimi-Mameghani et al., Iran, 2013 [15]	☆	☆	☆	☆	☆☆	☆	☆	☆
Basraon et al., USA, 2015 [14]	☆	☆	☆	☆	☆☆	☆	☆	☆
Han et al., China, 2017 [13]	☆	☆	☆	☆	☆☆	☆	☆	☆
Zhu et al., California, 2018 [18]	☆	☆	☆	☆	☆☆	☆	☆	☆
Zhu et al., California, 2018 [18]	☆	☆	☆	☆	☆	☆	☆	☆
Xu et al., China, 2016 [23]	☆	☆	☆	☆	☆☆	☆	☆	☆

The definition/explanation of each column of the NOS is available at http://www.ohri.ca/programs/clinical_epidemiology/oxford.asp.

**Table 3:** Summary result of the association between central obesity and GDM.

	Central obesity					
	No. of studies	OR	95% CI	*I* ^2^ (%)	*P*_h_^∗^	*P*_h_^∗^
Overall adjusted studies	11	2.76	2.35–3.26	14.4	0.307	
Subgroup analyses						
Geographical location						0.083
Asian	4	2.48	1.97–3.13	31.6	0.223	
Non-Asian	7	3.42	2.61–4.48	0.0	0.774	
No. of participants						0.083
<1000	6	3.66	2.61–5.16	0.0	0.755	
≥1000	5	2.56	2.12–3.09	23.6	0.264	
No. of cases						0.125
<100	7	3.44	2.53–4.66	0.0	0.766	
≥100	4	2.57	2.03–3.26	42.6	0.156	
Method of exposure						0.861
SFT	1	2.96	0.95–9.24	NA	NA	
WC/WHR	6	2.71	2.38–3.09	0.0	0.696	
BFI/FMP	2	2.79	1.18–6.59	81.5	0.020	
VAT	2	4.69	0.99–22.16	46.6	0.171	
Trimester						0.416
First	9	2.73	2.41–3.10	0.0	0.732	
Second	2	2.79	1.18–6.59	81.5	0.020	
Assessment of outcome						0.075
Carpenter and Coustan	5	2.51	2.14–2.95	10.2	0.348	
WHO	5	3.76	2.72–5.21	0.0	0.702	
Self-reported	1	3.00	1.08–8.34	NA	NA	
Adjustment for confounders						
Maternal age						0.951
Yes	10	2.80	2.33–3.36	22.7	0.234	
No	1	2.96	0.95–9.24	NA	NA	
Ethnicity						0.998
Yes	6	2.68	2.35–3.05	0.0	0.850	
No	5	3.22	1.91–5.42	48.6	0.047	
BMI						0.500
Yes	9	3.07	2.35–4.00	27.7	0.198	
No	2	2.58	2.24–2.98	0.0	0.940	
Family history of diabetes						0.415
Yes	6	2.74	2.40–3.12	0.0	0.498	
No	5	2.68	1.77–4.07	38.2	0.167	
Parity						0.103
Yes	6	2.57	2.21–2.99	7.7	0.367	
No	5	3.65	2.55–5.21	0.0	0.570	
Smoking						0.984
Yes	4	2.67	2.33–3.05	0.0	0.592	
No	7	3.01	2.10–4.33	38.6	0.134	
Education level						0.689
Yes	5	2.71	2.38–3.09	0.0	0.560	
No	6	2.90	1.91–4.41	40.6	0.135	

CI: confidence interval; NA: not available; RR: relative risk; SFT: subcutaneous fat thickness; WC/WHR: waist circumference/waist-to-hip ratio; BFI/FMP: body fat index/fat mass percentage; VAT: visceral adipose tissue depth; BMI: body mass index. ^∗^*P*-value for heterogeneity within each subgroup. ^∗∗^*P*-value for heterogeneity between subgroups in meta-regression analysis.

## Supplementary Materials

Supplementary Materials The search strategy in PubMed, Embase, and Web of Science of the relationship between “Central obesity” and “Gestational diabetes mellitus”.
