# Supplementary material for: Relationship between Maternal Central Obesity and the Risk of Gestational Diabetes Mellitus: A Systematic Review and Meta-Analysis of Cohort Studies
Source: J Diabetes Res. 2020 Apr 2;2020:6303820. doi: 10.1155/2020/6303820 (PMC7157762; doi:10.1155/2020/6303820)
Supplement: Supplementary Materials — The search strategy in PubMed, Embase, and Web of Science of the relationship between “Central obesity” and “Gestational diabetes mellitus”. [file 6303820.f1.pdf]

Supplement Table 1. The search strategy in PubMed, Embase and Web of Science of the relationship between "Central obesity" and "Gestational diabetes mellitus"

#1	Search "Obesity, Abdominal"[MeSH]/[exp]
#2	"Obesity, Abdominal" OR "Abdominal Obesities" OR "Obesities, Abdominal" OR "Abdominal Obesity" OR "Central Obesity" OR "Central Obesities" OR "Obesities, Central" OR "Obesity, Central" OR "Obesity, Visceral" OR "Visceral Obesity" OR "Obesities, Visceral" OR "Visceral Obesities"
#3	Search "Waist Circumference"[MeSH]/ [exp]
#4	"waist circuit" OR "waistline"
#5	Search "Waist-Hip Ratio"[MeSH]
#6	"Ratio, Waist-Hip" OR "Ratios, Waist-Hip" OR "Waist Hip Ratio" OR "Waist-Hip Ratios" OR "Waist-to-Hip Ratio" OR "Ratio, Waist-to-Hip" OR "Ratios, Waist-to-Hip" OR "Waist to Hip Ratio" OR "Waist-to-Hip Ratios"
#7	Search "Body Fat Distribution"[MeSH]/[exp]
#8	"Distribution, Body Fat" OR "Fat Distribution, Body" OR "Body Fat Patterning" OR "Fat Patterning, Body" OR "Patterning, Body Fat" OR "Body Fat Index"
#9	#2 OR #4 OR #6 OR #8
#10	Search "Pregnancy"[MeSH]/[exp]
#11	"gestation" OR "gestational" OR "pregnant women" OR "gravidity" OR "pregnancies"
#12	Search "Diabetes, Gestation"[MeSH]/[exp]
#13	"blood glucose" OR "glucose metabolism" OR "glucose tolerance" OR "glucose intolerance" OR "hyperglycemia" OR "gestational diabetes" OR "gestational diabetes mellitus" OR "pregnancy-induced diabetes" OR "diabetes, pregnancy-induced" OR "diabetes, pregnancy induced" OR "diabetes mellitus, gestational" OR "pregnancy in diabetes" OR "Diabetes"
#14	#9 AND #11 AND #13
